# Integrative Biomarker Panel for Improved Lung Cancer Diagnosis Using Plasma microRNAs and Sputum Bacterial DNA

**DOI:** 10.3390/curroncol31100444

**Published:** 2024-10-02

**Authors:** Pushpa Dhilipkannah, Ashutosh Sachdeva, Van K. Holden, Feng Jiang

**Affiliations:** 1 Department of Pathology, University of Maryland School of Medicine, Baltimore, MD 21201-1192, USA; 2 Department of Medicine, University of Maryland School of Medicine, Baltimore, MD 21201-1192, USAvholden@som.umaryland.edu (V.K.H.)

**Keywords:** diagnosis, early stage, lung cancer, plasma, biomarkers

## Abstract

This study aimed to evaluate if integrating diverse molecular biomarkers in plasma and sputum could improve the diagnosis of lung cancer. The study analyzed miRNAs in plasma and bacterial DNA in sputum from 58 lung cancer patients and 62 cancer-free smokers using droplet digital PCR. The individual plasma miRNA and sputum bacterial biomarkers had sensitivities of 62–71% and specificities of 61–79% for diagnosing lung cancer. A panel of plasma miRNA or sputum bacterial biomarkers produced sensitivities of 79–85% and specificities of 74–82%. An integromic signature consisting of two miRNAs in plasma and three bacterial biomarkers in sputum had a higher sensitivity (87%) and specificity (89%) compared to individual biomarkers. The signature’s diagnostic value was confirmed in a validation cohort of 56 lung cancer patients and 59 controls, independent of tumor stage, histological type, and demographic factors. Integrating diverse molecular biomarkers in plasma and sputum could improve the diagnosis of lung cancer.

## 1. Introduction

Non-small cell lung cancer (NSCLC) is the most common type of lung cancer, accounting for approximately 85% of all lung cancer cases. The incidence rate of NSCLC has been declining in recent years, primarily due to increased awareness of risk factors and advancements in treatment. However, lung cancer remains a significant public health concern, with an estimated 235,760 new cases of lung and bronchus cancers expected to be diagnosed in the United States in 2022 alone. NSCLC is a deadly disease, with the highest mortality rate of all cancers [1]. NSCLC mainly consists of two histological types: adenocarcinoma (AC) and squamous cell carcinoma (SCC). The survival rate for NSCLC varies from 10% to 56% depending on the stage at diagnosis. As the prognosis for patients with lung cancer is closely linked to the tumor stage, detecting lung cancer at an early or curable stage can significantly reduce mortality rates [1]. The early detection of lung cancer in a large randomized trial using low-dose CT (LDCT) has revealed a 20% relative reduction in mortality as compared to chest X-rays [1,2,3,4,5,6]. However, LDCT is associated with over-diagnosis, excessive cost, and radiation exposure [2,3,4,5,6]. The development of molecular biomarkers that can precisely diagnose early-stage NSCLC is required.

During tumor development, cancer cells undergo apoptosis and necrosis, and release tumor-associated molecules that can circulate in the bloodstream. The circulating tumor-derived molecules in plasma provide non-invasive cancer biomarkers. MicroRNAs (miRNAs) are small molecules and the aberrations contribute to carcinogenesis [7,8,9,10]. Using whole-transcriptome NGS, we have systematically and comprehensively characterized the miRNA profiles of lung tumors [11]. We have identified three miRNAs (miRs-126-3p, 205-5p, and 210-3p) as plasma biomarkers for NSCLC [11].

Sputum is another type of non-invasively obtained specimen that is secreted from the bronchi and bronchioles of the lower respiratory tract. Since molecular changes detected in sputum could reflect those in the lower respiratory tract [12], sputum can be substituted for the lower-airway fluids (e.g., bronchoalveolar lavage) and surgical tissues, which are more invasively collected for the detection of lung cancer [12]. It has been recently suggested that the microbiome plays crucial roles in tumor formation via direct mutagenesis, promoting the survival of tumor-initiating cells, or inducing pro-tumorigenic immune responses [4,5,6,7,8,9,10,11,12,13]. Of the microbes, bacteria have been studied in malignancies, including lung cancer [14]. We previously found that *Streptococcus* promoted lung tumorigenesis by triggering NF-kB pathways via binding PspC to PAFR [15]. We have recently shown that analysis of *Acidovorax, Capnocytophaga, Streptococus,* and *Veillonella* in sputum could improve the detection of NSCLC [8,9,10,11,12,13,14,15,16,17]. Therefore, microbiota might provide biomarkers for the early detection and diagnosis of lung cancer. 

Although the individual panels of plasma miRNA or sputum bacterial biomarkers show promise for lung cancer diagnosis, their sensitivities and specificities are not sufficient to be used in laboratory settings. One of the major reasons might be that NSCLC is a heterogeneous disease driven by multifactorial molecular aberrations; only one type of molecular change detected in one type of specimen may not achieve the diagnostic performance required in clinics. Furthermore, sputum has several limitations in terms of diagnosis, including insufficient specimen collection by patients and inadequate sensitivity and specificity. Thus, diagnostic tests such as blood tests may be required to improve the accuracy of sputum tests. In this study, we investigate if integrated analysis of the different types of molecular biomarkers in plasma and sputum can improve the early detection of lung cancer. 

## 2. Materials and Methods

### 2.1. Patients and Clinical Specimens

Using a protocol approved by the University of Maryland Baltimore Institutional Review Board, we recruited lung cancer patients and cancer-free smokers according to the inclusion and/or exclusion criteria recommended by U.S. Preventive Services Task Force. Briefly, we enrolled smokers between the ages of 50–80 who had at least a 20 pack-year smoking history and were former smokers (quit within 15 years). Exclusion criteria included pregnancy, current pulmonary infection, surgery within 6 months, radiotherapy within 1 year, and life expectancy of <1 year. The surgical-pathologic staging of NSCLC was used as the ground truth according to the TNM classification of the International Union Against Cancer (UICC) with the American Joint Committee on Cancer (AJCC) and the International Staging System for Lung Cancer. 

We collected blood in a BD Vacutainer spray-coated K2EDTA Tubes (BD, Franklin Lakes, NJ, USA) and prepared plasma using the standard operating protocols developed by the NCI-Early Detection Research Network. Briefly, the blood specimens were processed within one hour of collection by centrifugation at 1300× *g* for 10 min at 4 °C. Sputum was collected from the participants before they received any treatment as described in our previous studies [17,18,19,20,21,22,23,24,25,26,27]. The sputum samples were processed on ice in 4 volumes of 0.1% dithiothreitol (Sigma-Aldrich, St. Louis, MO, USA) followed by 4 volumes of phosphate-buffered saline (Sigma-Aldrich). We centrifuged the samples at 1500× *g* for 15 min and removed the supernatant. The remaining cell pellets were collected and stored at −80 °C until use. 

### 2.2. RNA Isolation and Droplet Digital PCR (ddPCR) Analysis of miRNAs

RNA was extracted from plasma by using a Trizol LS reagent (Invitrogen, Carlsbad, CA, USA) and RNeasy Mini Kit (Qiagen, Hilden, Germany). The qualification and quantification of RNA were assessed by using a Biospectrometer (Hutchinson Technology Inc., Hutchinson, MN, USA) and an Electrophoresis Bioanalyzer (Agilent Technologies, Foster City, CA, USA). Reverse Transcriptase (RT) was carried out to generate cDNA by using an RT Kit (Applied Biosystems, Foster City, CA, USA). ddPCR for analysis of expression levels of miRNAs was performed as described in our published works by using a QX200™ Droplet Digital™ PCR System (Bio-Rad, Hercules, CA, USA) [5,6,7,10,11,12,13,14,16,17,18,19,20,21,22,23,24,26,28]. Dynamic ranges and sensitivities of ddPCR for the detection of bacterial genera. Briefly, a PCR reaction mix containing cDNA was partitioned into aqueous droplets in oil via a QX100 Droplet Generator (Bio-Rad), and then transferred to a 96-well PCR plate. A two-step thermocycling protocol (95 °C × 10 min; 40 cycles of [94 °C × 30 s, 60 °C × 60 s], 98 °C × 10 min) was undertaken in a T100™ thermal cycler (Bio-Rad). The PCR plate was then transferred to the QX100 Droplet Reader for automatic reading of samples in all wells. The copy number of each gene per µL PCR reaction was directly determined. We used QuantaSoft 1.7.4 analysis software (Bio-Rad) and Poisson statistics to compute droplet concentrations (copies/μL). Only miRNAs that had at least 10,000 droplets were considered to be robustly detectable by ddPCR in plasma and subsequently underwent further analysis. All assays were done in triplicates, and one no-template control and two interplate controls were carried along in each experiment.

### 2.3. DNA Isolation and ddPCR Analysis of Bacterial Abundances

We used a QIAGEN-DNeasy Blood & Tissue Kit (Qiagen) to isolate DNA according to the manufacturer’s instructions. We determined the purity by taking the optical density (OD) of the sample at 280 nm for protein concentration and at 260 nm for DNA concentration. The ratio OD260/OD280 was calculated and the DNA sample within the range of 1.6–2 was considered as pure. We measured DNA copy numbers of bacterial genera by using the QX100 Droplet Digital PCR System and 2× ddPCR Supermix (Bio-Rad) with a protocol developed in our previous studies [8,9,10,11,12,13,14,15,16,17]. Dynamic ranges and sensitivities of ddPCR for the detection of bacterial genera were shown in the previous study [8,9,10,11,12,13,14,15,16,17]. To generate the droplets, we inserted 20 µL of PCR reaction and 70 µL of Droplet Generation oil for Probes (Bio-Rad) in an eight-well cartridge using a QX100 droplet generator (Bio-Rad). We then transferred 40 µL of the generated droplet emulsion in a 96-well PCR plate (Eppendorf, Hamburg, Germany). Amplification reaction was conducted in a T100™ thermal cycler (Bio-Rad) with the following conditions: initial denaturation at 95 °C for 5 min followed by 35 cycles of 15 s at 95.0 °C, 30 s at 55.3 °C, 5 min at 4 °C, and finally, 5 min at 90 °C for signal stabilization. After thermal cycling, we transferred plates to a droplet reader (Bio-Rad). We used the software provided with the ddPCR system for data acquisition to calculate the concentration of target DNA in copies/µL from the fraction of positive reactions using Poisson distribution analysis.

To ensure that the miRNA expression levels were reliable and reproducible across different samples, we included a non-human miRNA, cel-miR-39, as an exogenous control in both plasma and sputum samples. A synthetic cel-miR-39 RNA oligonucleotide (Integrated DNA Technologies, Inc., Coralville, IA, USA) was prepared at a concentration of 25 fmol in 5 µL of nuclease-free water and added to each plasma or sputum sample after the addition of a 2× denaturing solution to prevent degradation by endogenous RNases. Since ddPCR results are represented as copy numbers per microliter (copies/µL) instead of cycle threshold (Ct) values, we normalized miRNA expression to cel-miR-39 in each sample using the following method: First, we calculated a normalization factor for each sample using the copy number of cel-miR-39. This factor represents the efficiency and consistency of the RNA extraction and reverse transcription processes; Normalization factor = (copy number of cel-miR-39 in sample)/(average copy number of cel-miR-39 across all samples). To normalize the target miRNA, we adjusted the copy number of the target miRNA in each sample by dividing it by the normalization factor calculated in the previous step; Normalized miRNA copy number = (copy number of target miRNA)/(normalization factor). This approach ensured that the normalized miRNA expression levels were consistent and accurate across different samples, accounting for any technical variations in the RNA extraction and reverse transcription processes. 

### 2.4. Statistical Analysis

To determine sample size, we set the area under the curve (AUC) of H0 (the null hypothesis) at 0.5. H1 represented the alternative hypothesis. To have a high reproducibility with adequate precision, we required ≥28 subjects per group. With this sample size, we would have 85% power to detect an AUC of 0.75 at the 2% significance level. Therefore, the sample size in the two cohorts could have enough statistical power. Pearson’s correlation analysis was applied to assess the relationship of miRNA expressions or bacterial abundances with the demographic and clinical characteristics of the participants. AUCs were used to determine accuracy, sensitivity, and specificity of each gene. We used the highest Youden’s J index (sum of sensitivity and specificity-1) to set up the corresponding cut-off value. We employed multivariate logistic regression models to adjust for potential confounding variables, such as age, gender, smoking history, and comorbidities, in the biomarker analysis. By adjusting for these factors, we isolated the true association between the biomarkers and lung cancer diagnosis. Logistic regression models with constrained parameters as in LASSO were used to eliminate the irrelevant factors, develop composite panels of biomarkers, and optimize a signature with the highest sensitivity and specificity. To compare the signature and our previously developed plasma and sputum biomarker panels, we compared their AUCs to determine the sensitivity, specificity, and accuracy as previously described.

## 3. Results

### 3.1. The Demographic and Clinical Variables of Cases and Controls 

A total of 114 NSCLC patients and 121 cancer-free smokers were recruited. Among the cancer patients, 39 patients were female and 75 were male. Fifty-six had stage I NSCLC, 33 with stage II, 15 with stage III, and 10 with stage IV. Sixty-five lung cancer patients were diagnosed with AC, while 49 with SCC. Of the cancer-free smokers, 39 patients were female and 75 were male. There were no significant differences in age, gender, and smoking status between the NSCLC patients and cancer-free smokers. The cases and controls were randomly grouped into two cohorts: a development cohort and a validation cohort. The development cohort consisted of 58 lung cancer patients and 62 cancer-free smokers, while the validation cohort comprised 56 lung cancer patients and 59 cancer-free smokers. The demographic and clinical variables of the two cohorts are shown in Supplementary Tables S1 and S2.

### 3.2. The Diagnostic Performance of Plasma miRNA and Sputum Bacterial Biomarkers in Distinguishing between Lung Cancer Patients and Cancer-Free Controls

**Figure 1:**
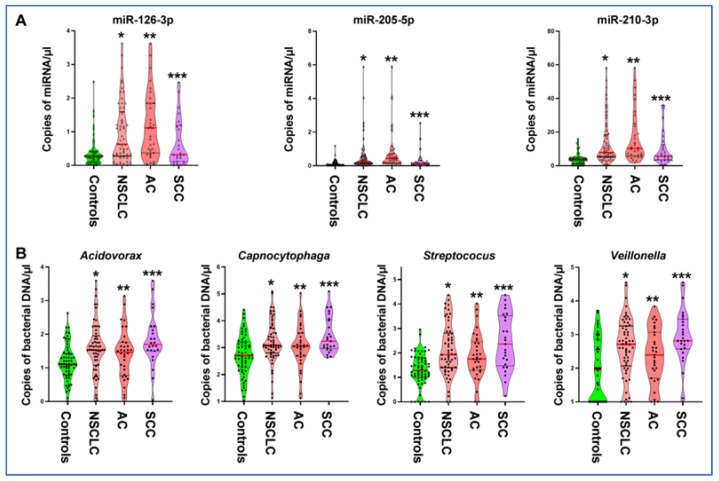
miRNAs in plasma and bacteria in sputum of 58 lung cancer patients and 62 controls. (**A**) Plasma miRNAs displayed a higher level in NSCLC patients compared with control individuals (*, all *p* < 0.05). The miRNAs show a higher level in AC patients compared with SCC patients (**, all *p* < 0.05). The miRNAs show a higher level in SCC patients compared with cancer-free smokers (***, all *p* < 0.05). (**B**) Sputum bacteria genera have higher DNA abundances in NSCLC patients compared with control individuals (*, all *p* < 0.05). Sputum bacteria genera display a higher level in AC patients compared with cancer-free smokers (**, all *p* < 0.05). Sputum bacteria genera show a higher level in SCC patients compared with AC patients (***, all *p* < 0.05).

ddPCR analysis of miRNAs in plasma and bacteria in the sputum of a development cohort generated at least 10,000 droplets in each well of the plates. Therefore, miRNA expression and bacterial DNA abundances could be ‘‘read’’ by ddPCR for their absolute quantification in the clinical samples. The plasma miRNAs displayed a different level in 58 NSCLC patients compared with 62 control individuals (all *p* ≤ 0.001) (Figure 1A) (Table 1). As a result, the individual plasma miRNA had an AUC of 0.70–0.81, producing 68.97% to 77.59% sensitivities and 66.63% to 78.69% specificities for detection of NSCLC (Table 1). Furthermore, the plasma miRNA biomarkers were significantly associated with AC (All *p* ≤ 0.05) (Figure 1A). In addition, combined use of the three plasma miRNAs as a panel of biomarkers created 79.31% sensitivity, 85.48% specificity, and 82.50% accuracy for diagnosis of NSCLC (Table 2). Since the plasma miRNAs were associated with histological types, the panel of three plasma miRNA biomarkers had a higher diagnostic value for AC with 81.82% sensitivity, 85.48% specificity, and 84.21% accuracy compared with SCC (72.00% sensitivity, 83.87% specificity, and 80.46% accuracy, all *p* < 0.05) (Table 2). However, the plasma miRNA biomarkers did not have a relationship with the stage of the NSCLC and age, race, and sex of the participants (All *p* ≥ 0.05), except their smoking history (*p* < 0.05) (Supplementary Table S3). Altogether, the plasma miRNAs could have the potential as biomarkers for early-stage NSCLC, particularly lung AC.

In the same development cohort, bacterial genera tested in sputum exhibited a higher level of DNA abundance in NSCLC patients compared with cancer-free smokers (All *p* ≤ 0.001) (Figure 1B) (Table 1). The individual bacterial genera created 0.66–0.73 AUC with 62.07–70.86% sensitivities and 61.29–79.03% specificities for diagnosis of NSCLC (Table 1) (Supplementary Figure S1). DNA abundance of the four bacterial genera in sputum was more related to SCC compared with AC (*p* < 0.05) (Figure 1B). The use of the four bacteria genera in combination produced a higher AUC compared with each bacterium used alone (Supplementary Figure S1). As a result, combined use of the four bacteria created 68.97% sensitivity, 79.03% specificity, and 74.17% accuracy for the diagnosis of NSCLC, and was significantly higher than each one used alone (Table 1 and Table 2). The panel of sputum bacterial biomarkers had a higher diagnostic value for SCC with 76.00% sensitivity, 85.48% specificity, and 82.76% accuracy compared with AC (66.67% sensitivity, 72.58% specificity, and 70.52% accuracy, all *p* < 0.05) (Table 2). The sputum bacterial biomarkers were not associated with the stage of NSCLC, and age, sex, and ethnicity of the participants (All *p* ≥ 0.05), except their smoking history (*p* < 0.05) (Supplementary Table S3). Therefore, the sputum bacterial biomarkers had the potential as biomarkers for early-stage NSCLC, especially lung SCC.

### 3.3. Combined Use of Plasma miRNA and Sputum Bacterial Biomarkers for Early Detection of NSCLC 

**Figure 2:**
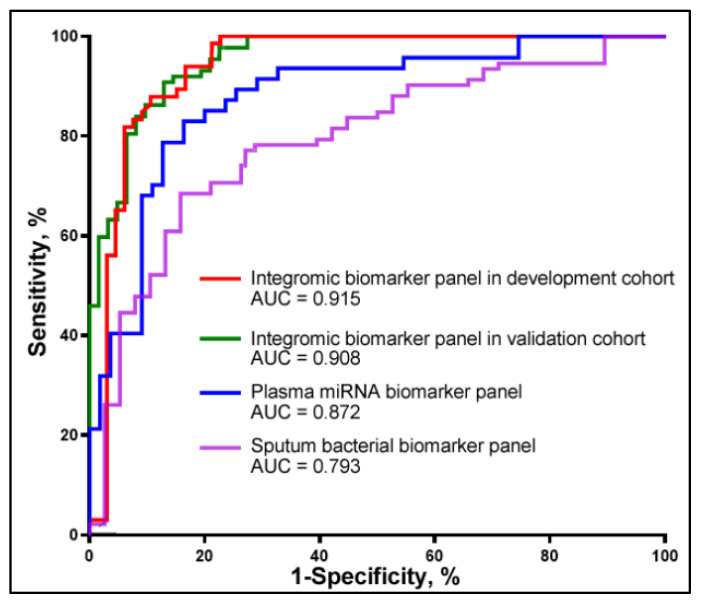
Diagnostic performance of plasma miRNA and sputum bacterial biomarkers for lung cancer. The red and green lines show the performance of the integromic biomarker panel in the development cohort and validation cohort, respectively. There is no statistically significant difference between the AUCs of the integromic biomarker panel in the development cohort versus the validation cohort (*p* = 0.84). The blue and purple lines represent the diagnostic performance of the three-plasma miRNA biomarker panel and the four-sputum bacterial biomarker panel, respectively, in the development cohort.

We further investigated if integrated analysis of the two classes of molecular changes in plasma and sputum might have a synergetic effect on the detection of lung cancer. We used least absolute shrinkage and selection operator (LASSO) to identify and optimize a panel of biomarkers for lung cancer. Two plasma miRNAs (miRs-210-3p and -205-5p) and three sputum bacteria (*Acidovorax*, *Streptococus*, and *Veillonella*) biomarkers were selected as the best ones (all *p* < 0.001) and integrated into a logistic model. Integrated use of the five biomarkers yielded a greater AUC (0.91) than did the three-plasma miRNA biomarker panel (0.87) or the four-sputum bacterial biomarker panel (0.79) (All *p* < 0.05) (Figure 2). We used the highest Youden’s J index to set up the corresponding cut-off value. The optimal cut-off for the integromic biomarker panel was U = 1.27. Any subject with U ≥ 1.27 was classified as a lung cancer case. As a result, the integromic biomarker panel produced significantly higher sensitivity (84.48%), specificity (90.32%), and accuracy (87.50%) compared with the individual panels of biomarkers (all *p* < 0.05) (Table 2). The integromic biomarker panel could detect the positive cases that were positive by either plasma miRNA biomarkers or sputum bacterial biomarkers. Therefore, the integromic biomarker panel picked up the positive lung cancer cases identified by either the sputum biomarker panel or the plasma biomarker panel alone. Furthermore, the integrated analysis of diverse biomarkers across different body fluids showed no significant association with lung cancer stage, histology, or patients’ age, race, or gender (all *p* > 0.05). Additionally, no significant correlation was observed between plasma miRNAs and bacterial genera (all *p* > 0.05) (Supplementary Table S4).

### 3.4. Validation of the Integromic Biomarker Panel in a Different Cohort for the Diagnosis of NSCLC

The three plasma miRNA and four sputum bacterial biomarkers included in the integromic biomarker panel were assessed in a validation cohort of 56 NSCLC patients and 59 cancer-free smokers. Combined analysis of the miRNAs and bacterial genera using the logistic regression model created 0.90 AUC for lung cancer diagnosis. There was no significant difference between the development cohort and validation cohort regarding the AUCs (0.91 vs. 0.90, *p* = 0.79) (Figure 2). The biomarkers used in combination could differentiate the NSCLC patients from cancer-free smokers with 87.50% sensitivity, 89.83% specificity, and 88.70% accuracy. In line with the findings in the development cohort, the integromic biomarker panel was independent of stage and subtype of NSCLC (all *p* > 0.05). Moreover, there was no association of the molecular changes with age, race, and gender (All *p* > 0.05), except with the smoking status of the individuals (*p* < 0.05). 

## 4. Discussion

Given that altered miRNAs and pathogenic bacteria contribute to lung tumorigenesis via different mechanisms, we hypothesize that integrating the two types of molecular changes in different body fluids may have a synergetic effect on the diagnosis of lung cancer. Our results show that the combined analysis of miRNAs in plasma and bacterial genera in sputum yielded a better performance than does a single category of the biomarkers, and thus validates the hypothesis. Furthermore, unlike plasma miRNA markers that are more specific to AC and sputum bacterial biomarkers that are more specific to SCC, combined analysis of the plasma and sputum biomarkers is independent of the histology of NSCLC, and hence substantiates the utility for predicting lung cancer. In addition, the integromic biomarker panel has a comparable diagnostic performance for lung tumor at the early versus late stages. Furthermore, current screening tests lack effectiveness in detecting lung cancer in smoking populations, where the risk is higher. The results show that combining miRNAs in plasma and bacterial genera in sputum has superior diagnostic performance compared to using a single category of biomarkers. The discovery of the integromic biomarker panel would be important in the early detection of NSCLC in smokers.

Dysregulations of miRs-126-3p, 205-5p, and 210-3p have been consistently associated with various tumors, including NSCLC, as reported in previous studies. For example, miR-126-3p has been shown to constrain cell proliferation in NSCLC by downregulating its target, EGFL7 [19,20,21,22,23,24,25]. In line with prior findings, our study also observed elevated levels of miR-126-3p in plasma from NSCLC patients, supporting its established role in lung carcinogenesis. Regarding miR-205-5p, earlier research has highlighted its higher expression in lung tumor tissues compared to non-cancerous lung tissues, positioning it as a potential biomarker for NSCLC [6,22,28]. Moreover, the upregulation of miR-205-5p has been shown to contribute to lung cancer cell proliferation and metastasis by regulating TP53INP1, RB1, and P21 [26]. Our findings are in agreement with these studies, and we reinforce the role of miR-205-5p as a biomarker for NSCLC. However, our study adds to the literature by demonstrating that miR-205-5p is also detectable in plasma, offering a non-invasive diagnostic marker. miR-210-3p has similarly been implicated in the diagnosis of various malignancies, including lung cancer [28,29,30,31,32,33]. Our observation of elevated miR-210-3p in plasma samples from NSCLC patients confirms its relevance in this context. Although miR-210-3p has been reported in other NSCLC studies, its diagnostic potential in plasma has not been fully explored. Our study contributes novel evidence supporting its use as a non-invasive biomarker for NSCLC diagnosis. Altogether, while miRs-126-3p, 205-5p, and 210-3p have been previously identified as dysregulated in NSCLC, the novelty of our study lies in their detection in plasma as non-invasive biomarkers. Moreover, this study provides an integrated analysis combining miRNA and bacterial biomarkers, which has not been extensively explored in previous studies. Our findings are in consensus with previous research in terms of miRNA dysregulation, but we extend the understanding of their diagnostic utility in a non-invasive context.

Among the bacterial genera analyzed, *Acidovorax* was found by Greathouse et al. to have an elevated abundance in lung tumor tissues with TP53 mutation [34]. Furthermore, there was a significant increase in lung tumor volume in mice inoculated with *Acidovorax*. *Acidovorax* could contribute to lung carcinogenesis in the presence of activated Kras and mutant p53, and thus act as a promoter in the development and progression of the disease [34]. *Capnocytophaga* was proposed to be involved in lung carcinogenesis and lower respiratory tract infections [35]. Furthermore, *Capnocytophaga* might induce long-term immune response/infection to the organ or cancer growth environment, which favors the growth of these bacteria in the airways [36]. We previously showed that *Streptococcus* attached to tumor cells by binding PspC to PAFR [15]. *Streptococcus* may have a crucial function in carcinogenesis via triggering the PI3K/AKT and NF-kB pathways. *Veillonella* was found to be over-represented in the lower airways of patients with NSCLC and related with ERK and PI3K signaling dysregulation [27]. Our current research suggests that *Veillonella* could serve as a potential biomarker for NSCLC [8,9,10,11,12,13,14,15,16,17]. However, further studies are required to confirm the finding.

The relationship between the lung microbiome and cancer development is complex, with emerging research highlighting how microbial communities influence cancer pathogenesis. The lung microbiome can modulate immune responses, with certain bacteria either enhancing anti-tumor activity or promoting immune evasion by upregulating regulatory T cells (Tregs) or dampening cytotoxic T-cell function. Additionally, microbial dysbiosis can influence the tumor microenvironment by altering cell signaling pathways, angiogenesis, and metabolic processes. Microbiota-driven changes in epithelial integrity and oxidative stress can lead to genetic mutations, while some bacteria may facilitate carcinogen metabolism, increasing lung cancer risk. These findings underscore the microbiome’s role in lung cancer pathogenesis and its potential as a target for novel therapeutic strategies.

The study does have limitations. First, the sample size of the cohorts is small. We will perform a new study to prospectively validate the biomarkers for lung cancer early detection using a large population. Second, various factors can impact the microbiome and hinder the identification of cancer biomarkers, including COPD, diet, smoking, and occupational exposure. As these factors were not available in the current project, we cannot determine if the identified biomarkers are associated with confounding factors. A new study will be conducted, selecting participants with minimal exposure to occupational hazards and gathering detailed information on their diet and lifestyle to control variables. A subgroup analysis will also evaluate the impact of COPD on the biomarkers’ diagnostic performance. Third, we only evaluate three miRNAs and four bacterial genera to develop this integromic biomarker panel. The 87.50% sensitivity and 89.83% specificity of the integromic panel are not sufficient to be used in the clinics. To address the limitation, we are evaluating additional miRNAs and bacteria that are associated with NSCLC to develop more biomarkers to further improve the performance of the diagnostic approach. Furthermore, cell-free tumor DNA (ctDNA) or methylation profiles of ctDNA, have been suggested as potential biomarkers for lung cancer diagnostics and prognostics. We will compare the miRNA and bacterial DNA biomarkers with ctDNA biomarkers and investigate if their combination could provide a better diagnostic value for lung cancer. Fourth, the relationship between cancer and the microbiome is complex and not fully understood. We will investigate if NSCLC leads to an overgrowth of certain strains of bacteria and identify potential therapeutic targets for improving cancer outcomes by modulating the microbiota. Fifth, we have demonstrated that circulating bacterial DNA can serve as plasma biomarkers for early lung cancer detection [34,35,36]. In the future, we plan to compare the combined analysis of miRNAs in plasma and bacterial genera in sputum with bacterial DNA in plasma to assess and enhance their diagnostic performance.

## 5. Conclusions

Given the heterogeneous nature of NSCLC developed from multifactorial molecular aberrations, we have for the first time demonstrated that the integration of miRNA and bacterial biomarkers across plasma and sputum could provide an efficient approach for the diagnosis of lung cancer. Nonetheless, a large multicenter clinical project to prospectively validate the full utility of the signature is required. 

## Figures and Tables

**Table 1:** Plasma miRNAs and sputum bacteria genera in a development cohort of 58 lung cancer patients and 62 cancer-free smokers.

miRNAs or Bacteria	Mean (SD) in Cancer-Free Smokers	Mean (SD) NSCLC	*p*	AUC (95% CI)	Sensitivity %	Specificity %
miR-126-3p	0.269 (0.419)	0.608 (1.393)	0.0003	0.702 (0 0.606 to 0.798)	68.97	66.63
miR-205-5p	0.0729 (0.183)	0.2276 (1.013)	0.0001	0.812 (0.735 to 0.889)	69.26	82.26
miR-210-3p	3.700 (2.697)	7.802 (13.689)	<0.001	0.806 (0.726 to 0.885)	77.59	78.69
*Acidovorax*	1.106 (0.537)	1.572 (0.786)	0.0010	0.680 (0.581 to 0.779)	62.07	79.03
*Capnocytophaga*	3.070 (0.957)	3.667 (0.737)	0.0009	0.656 (0.558 to 0.756)	63.79	61.29
*Streptococus*	1.312 (0.662)	2.111 (1.065)	<0.0001	0.729 (0.639 to 0.820)	70.86	54.29
*Veillonella*	1.010 (0.436)	1.778 (0.737)	0.0004	0.678 (0.579 to 0.776)	68.97	58.06

Abbreviations: NSCLC, non-small cell lung cancer, pulmonary nodules; SD, standard deviation; AUC, the area under receiver operating characteristic.

**Table 2:** Diagnostic performance of individual plasma miRNA and sputum bacteria biomarker panel and integromic biomarker panel in development cohort.

	Sensitivity	Specificity	Accuracy	Sensitivity	Specificity	Accuracy	Sensitivity	Specificity	Accuracy
Three-plasma miRNAs biomarker panel	81.82%	85.48%	84.21%	72.0%	83.87%	80.46%	79.31%	85.48%	82.50%
Four-sputum bacterial biomarker panel	66.67%	72.58%	70.52	76.0%	85.48%	82.76%	68.97%	79.03%	74.17%
The integromic biomarker panel	84.85%	90.32%	88.42%	84.0%	90.32%	88.51%	84.48%	90.32%	87.50%

## Data Availability

The data that support the findings of this study are available from the corresponding author upon a reasonable request.

## Supplementary Materials

The following supporting information can be downloaded at: https://www.mdpi.com/article/10.3390/curroncol31100444/s1, Supplementary Table S1. Characteristics of a development cohort of NSCLC patients and cancer-free smokers; Supplementary Table S2. Characteristics of a validation cohort of NSCLC patients and cancer-free smokers; Supplementary Table S3. The association of changes of miRNAs and bacterial genera with the age, gender, ethnic group, and tumor stage, and smoking status of the patients determined by Pearson’s correlation coefficient test. A *p*-value < 0.05 is statistically significant; Supplementary Table S4. The association between plasma miRNAs and sputum bacterial biomarkers. Supplementary Figure S1. Combining four bacterial genera produced a higher AC compared with each bacterium.

## References

[1] Aberle D.R., Adams A.M., Berg C.D., Black W.C., Clapp J.D., Fagerstrom R.M., Gareen I.F., Gatsonis C., Marcus P.M., Sicks J.D. (2011). Reduced lung-cancer mortality with low-dose computed tomographic screening. N. Engl. J. Med..

[2] Wilson D.O. (2014). Lung cancer screening with low-dose CT (LDCT) is ready for prime time in the USA. Evid. Based Med..

[3] Marcus P.M. (2015). Lung cancer screening with low dose computed tomography (LDCT): Looking back and moving forward. Ann. Transl. Med..

[4] Oudkerk M., Liu S., Heuvelmans M.A., Walter J.E., Field J.K. (2021). Lung cancer LDCT screening and mortality reduction - evidence, pitfalls and future perspectives. Nat. Rev. Clin. Oncol..

[5] Pelosi G., Sonzogni A., Veronesi G., De Camilli E., Maisonneuve P., Spaggiari L., Manzotti M., Masullo M., Taliento G., Fumagalli C. (2008). Pathologic and molecular features of screening low-dose computed tomography (LDCT)-detected lung cancer: A baseline and 2-year repeat study. Lung Cancer.

[6] Wang X., Liu H., Shen Y., Li W., Chen Y., Wang H. (2018). Low-dose computed tomography (LDCT) versus other cancer screenings in early diagnosis of lung cancer: A meta-analysis. Medicine.

[7] Hashemi M., Khosroshahi E.M., Chegini M.K., Abedi M., Matinahmadi A., Hosnarody Y.S.D., Rezaei M., Saghari Y., Fattah E., Abdi S. (2023). miRNAs and exosomal miRNAs in lung cancer: New emerging players in tumor progression and therapy response. Pathol. Res. Pract..

[8] Hajipour S., Hosseini S.M., Irani S., Tavallaie M. (2023). Identification of novel potential drugs and miRNAs biomarkers in lung cancer based on gene co-expression network analysis. Genomics Inform..

[9] Braga E.A., Fridman M.V., Burdennyy A.M., Loginov V.I., Dmitriev A.A., Pronina I.V., Morozov S.G. (2023). Various LncRNA Mechanisms in Gene Regulation Involving miRNAs or RNA-Binding Proteins in Non-Small-Cell Lung Cancer: Main Signaling Pathways and Networks. Int. J. Mol. Sci..

[10] Zhang X., Tan J., Chen Y., Ma S., Bai W., Peng Y., Shi G. (2022). Identification of serum MiRNAs as candidate biomarkers for non-small cell lung cancer diagnosis. BMC Pulm. Med..

[11] Ma J., Mannoor K., Gao L., Tan A., Guarnera M.A., Zhan M., Shetty A., Stass S.A., Xing L., Jiang F. (2014). Characterization of microRNA transcriptome in lung cancer by next-generation deep sequencing. Mol. Oncol..

[12] Li R., Todd N.W., Qiu Q., Fan T., Zhao R.Y., Rodgers W.H., Fang H.B., Katz R.L., Stass S.A., Jiang F. (2007). Genetic deletions in sputum as diagnostic markers for early detection of stage I non-small cell lung cancer. Clin. Cancer Res..

[13] Garrett W.S. (2015). Cancer and the microbiota. Science.

[14] Glyn T., Purcell R. (2022). Circulating Bacterial DNA: A New Paradigm for Cancer Diagnostics. Front. Med..

[15] Li N., Zhou H., Holden V.K., Deepak J., Dhilipkannah P., Todd N.W., Stass S.A., Jiang F. (2023). Streptococcus pneumoniae promotes lung cancer development and progression. iScience.

[16] Mao Q., Jiang F., Yin R., Wang J., Xia W., Dong G., Ma W., Yang Y., Xu L., Hu J. (2018). Interplay between the lung microbiome and lung cancer. Cancer Lett.

[17] Zhou H., Liao J., Leng Q., Chinthalapally M., Dhilipkannah P., Jiang F. (2023). Circulating Bacterial DNA as Plasma Biomarkers for Lung Cancer Early Detection. Microorganisms.

[18] Leng Q., Holden V.K., Deepak J., Todd N.W., Jiang F. (2021). Microbiota Biomarkers for Lung Cancer. Diagnostics.

[19] Shi H., Bi H., Sun X., Dong H., Jiang Y., Mu H., Li W., Liu G., Gao R., Su J. (2018). Tubeimoside-1 inhibits the proliferation and metastasis by promoting miR-126-5p expression in non-small cell lung cancer cells. Oncol. Lett..

[20] Huang B., Wu G., Peng C., Peng X., Huang M., Ding J., Zhang H., Wu X. (2023). miR-126 regulates the proliferation, migration, invasion, and apoptosis of non-small lung cancer cells via AKT2/HK2 axis. IUBMB Life.

[21] Zheng W., Zhou Y., Lu J., Xu H., Lei L., Chen C., Zhao J., Xu L. (2017). The prognostic value of miR-126 expression in non-small-cell lung cancer: A meta-analysis. Cancer Cell Int..

[22] Kim M.K., Jung S.B., Kim J.S., Roh M.S., Lee J.H., Lee E.H., Lee H.W. (2014). Expression of microRNA miR-126 and miR-200c is associated with prognosis in patients with non-small cell lung cancer. Virchows. Arch..

[23] Zhu X., Li H., Long L., Hui L., Chen H., Wang X., Shen H., Xu W. (2012). miR-126 enhances the sensitivity of non-small cell lung cancer cells to anticancer agents by targeting vascular endothelial growth factor A. Acta. Biochim. Biophys Sin..

[24] Liu B., Peng X.C., Zheng X.L., Wang J., Qin Y.W. (2009). MiR-126 restoration down-regulate VEGF and inhibit the growth of lung cancer cell lines in vitro and in vivo. Lung Cancer.

[25] Zhang Z., Wang J., Cheng J., Yu X. (2018). Effects of miR-126 on the STAT3 signaling pathway and the regulation of malignant behavior in lung cancer cells. Oncol. Lett..

[26] Zhao Y.L., Zhang J.X., Yang J.J., Wei Y.B., Peng J.F., Fu C.J., Huang M.H., Wang R., Wang P.Y., Sun G.B. (2022). MiR-205-5p promotes lung cancer progression and is valuable for the diagnosis of lung cancer. Thorac. Cancer.

[27] Tsay J.J., Wu B.G., Badri M.H., Clemente J.C., Shen N., Meyn P., Li Y., Yie T.A., Lhakhang T., Olsen E. (2018). Airway Microbiota Is Associated with Upregulation of the PI3K Pathway in Lung Cancer. Am. J. Respir. Crit. Care Med..

[28] Chen Q., Zhang H., Zhang J., Shen L., Yang J., Wang Y., Ma J., Zhuan B. (2021). miR-210-3p Promotes Lung Cancer Development and Progression by Modulating USF1 and PCGF3. Onco. Targets Ther..

[29] Chen Q., Xie X. (2021). Association of Exosomal miR-210 with Signaling Pathways Implicated in Lung Cancer. Genes.

[30] Eilertsen M., Andersen S., Al-Saad S., Richardsen E., Stenvold H., Hald S.M., Al-Shibli K., Donnem T., Busund L.T., Bremnes R.M. (2014). Positive prognostic impact of miR-210 in non-small cell lung cancer. Lung Cancer.

[31] Fan J., Xu G., Chang Z., Zhu L., Yao J. (2020). miR-210 transferred by lung cancer cell-derived exosomes may act as proangiogenic factor in cancer-associated fibroblasts by modulating JAK2/STAT3 pathway. Clin. Sci..

[32] He R.Q., Cen W.L., Cen J.M., Cen W.N., Li J.Y., Li M.W., Gan T.Q., Hu X.H., Chen G. (2018). Clinical Significance of miR-210 and its Prospective Signaling Pathways in Non-Small Cell Lung Cancer: Evidence from Gene Expression Omnibus and the Cancer Genome Atlas Data Mining with 2763 Samples and Validation via Real-Time Quantitative PCR. Cell Physiol. Biochem..

[33] Hu X., Peng Q., Zhu J., Shen Y., Lin K., Shen Y., Zhu Y. (2020). Identification of miR-210 and combination biomarkers as useful agents in early screening non-small cell lung cancer. Gene.

[34] Greathouse K.L., White J.R., Vargas A.J., Bliskovsky V.V., Beck J.A., von Muhlinen N., Polley E.C., Bowman E.D., Khan M.A., Robles A.I. (2018). Interaction between the microbiome and TP53 in human lung cancer. Genome Biol..

[35] Yan X., Yang M., Liu J., Gao R., Hu J., Li J., Zhang L., Shi Y., Guo H., Cheng J. (2015). Discovery and validation of potential bacterial biomarkers for lung cancer. Am. J. Cancer Res..

[36] Gomes S., Cavadas B., Ferreira J.C., Marques P.I., Monteiro C., Sucena M., Sousa C., Vaz Rodrigues L., Teixeira G., Pinto P. (2019). Profiling of lung microbiota discloses differences in adenocarcinoma and squamous cell carcinoma. Sci. Rep..

