# Supplementary material for: Integrative Biomarker Panel for Improved Lung Cancer Diagnosis Using Plasma microRNAs and Sputum Bacterial DNA
Source: Curr Oncol. 2024 Oct 2;31(10):5949–59. doi: 10.3390/curroncol31100444 (PMC11506187; doi:10.3390/curroncol31100444)
Supplement: Supplementary file 1 [file curroncol-31-00444-s001.zip › curroncol-3146667-supplementary.pdf]

Supplementary files

Supplementary Table S1. Characteristics of a development cohort of NSCLC patients and cancer-free smokers

	NSCLC cases (n = 58)	Controls (n = 62)	P-value
Age	65.84 (SD 11.03)	64.27 (SD 11.27)	0.29
Sex			0.32
Female	20	23	
Male	38	39	
Race			0.34
African Americans	19	22	
White Americans	39	40	
Smoking pack-years (median)	33.7	30.5	0.13
Stage			
Stage I	28		
Stage II	17		
Stage III	8		
Stage IV	5		
Histological type			
Adenocarcinoma	33		
Squamous cell carcinoma	25		

Abbreviations: NSCLC, non-small cell lung cancer. SD, standard deviation.

Supplementary Table S2. Characteristics of a validation cohort of NSCLC patients and cancer-free smokers

	NSCLC cases (n = 56)	Controls (n = 59)	P-value
Age	65.84 (SD 11.03)	63.85 (SD 10.63)	0.27
Sex			0.32
Female	19	23	
Male	37	36	
Race			0.36
African Americans	18	19	
White Americans	38	40	
Smoking pack-years (median)	34.8	31.9	0.19
Stage			
Stage I	28		
Stage II	16		
Stage III	7		
Stage IV	5		
Histological type			
Adenocarcinoma	32		
Squamous cell carcinoma	24		

Abbreviations: NSCLC, non-small cell lung cancer. SD, standard deviation.

Supplementary Table S3. The association of changes of miRNAs and bacterial genera with the age, gender, ethnic group, and tumor stage, and smoking status of the patients determined by Pearson's correlation coefficient test. A p-value < 0.05 is statistically significant.

Genera	Age Correlation coefficients, P-value	Gender Correlation coefficients, P-value	Race Correlation coefficients, P-value	Smoking status Correlation coefficients, P-value	Stage Correlation coefficients, P-value
miR-126-3p	-0.3486	-0.8437	-0.3087	0.6520	-0.7327
	0.3779	0.2543	0.7863	**0.0344**	0.9953
miR-205-5p	-0.4586	0.2633	-0.3257	0.4430	-0.3237
	0.2868	0.5413	0.5853	**0.0163**	0.2863
miR-210-3p	-0.6216	-0.8663	0.5653	0.1050	0.2543
	0.7983	0.5523	0.2733	**0.0289**	0.6903
Acidovorax	-1.329	-0.1657	-0.4037	0.7410	-0.9127
	0.3893	0.5623	0.3643	**0.03333**	0.3843
Capnocytophaga	-0.4528	0.2633	-0.3257	0.4431	-0.3237
	0.2883	0.5413	0.5853	**0.0235**	0.2863
Streptococcus	-0.5145	-0.4447	-0.6447	0.6410	-0.7237
	0.7563	0.7763	0.8873	**0.0323**	0.7653
Veillonella	-0.6632	-0.9657	-0.7537	0.1217	0.9943
	0.1376	0.2533	0.2543	**0.0355**	0.3643

Supplementary Table S4. The assoaition between plasma miRNAs and sputum bacterial biomarkers.

Plasma miRNA / Bacterial Biomarker	*Streptococcus* (r, p-value)	*Veillonella* (r, p-value)	*Acidovorax* (r, p-value)	*Capnocytophaga* (r, p-value)
miR-126-3p	0.08, 0.580	-0.05, 0.700	0.12, 0.460	-0.10, 0.520
miR-205-5p	-0.03, 0.820	0.07, 0.620	0.06, 0.680	-0.04, 0.740
miR-210-3p	0.09, 0.540	-0.02, 0.870	0.11, 0.490	0.05, 0.720

The correlation coefficient (r) values are close to 0, indicating weak or no correlation. All p-values are greater than 0.05, signifying that no correlations between the biomarkers are statistically significant.

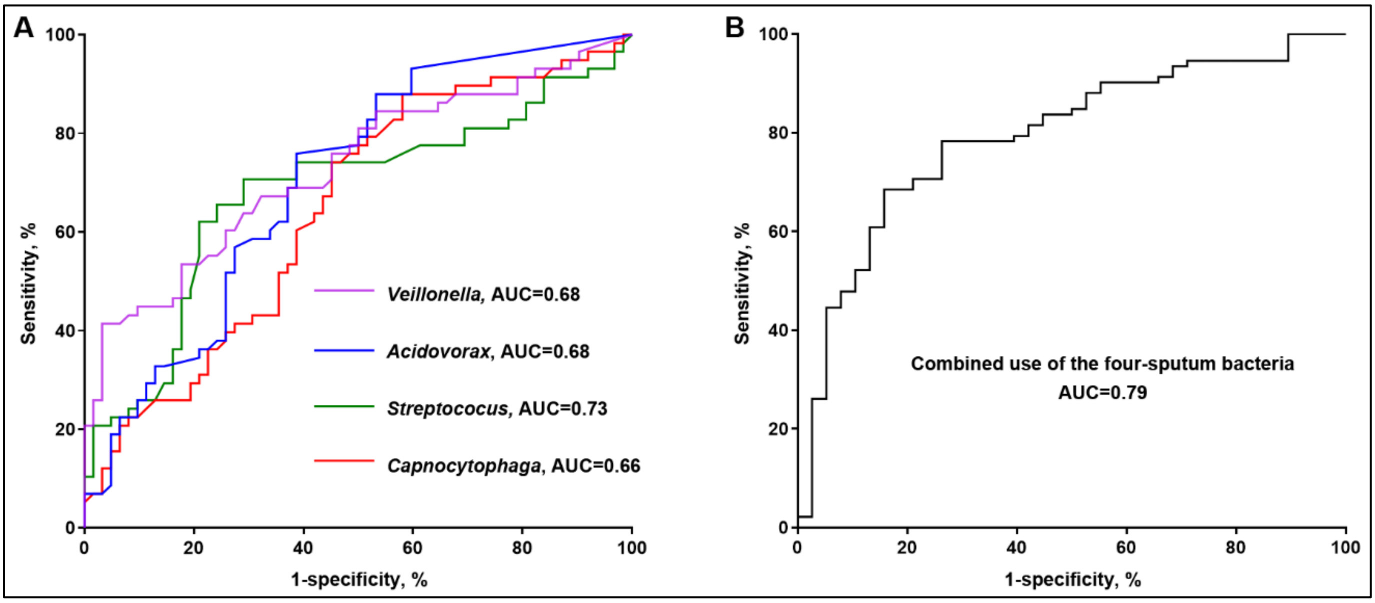

Supplementary Figure S1. Combining four bacterial genera produced a higher AUC compared to each bacterium individually.

ROC curve analysis was conducted on four bacterial genera in 58 lung cancer patients and 62 control individuals. The AUC values were calculated to assess the accuracy of each one in differentiating lung cancer patients from cancer-free individuals. **A.** Individually, the four bacterial genera produced AUC values of 0.66-0.73. **B.** When combined, the AUC value increased to 0.79, indicating higher accuracy in differentiating between the two groups.
